# The Effect of Alginate Concentration on Crystallinity, Morphology, and Thermal Stability Properties of Hydroxyapatite/Alginate Composite

**DOI:** 10.3390/polym15030614

**Published:** 2023-01-25

**Authors:** Wulandari Wulandari, Dini Muthiah Islami, Diana Vanda Wellia, Emriadi Emriadi, Vivi Sisca, Novesar Jamarun

**Affiliations:** 1 Department of Chemistry, University of Andalas, Padang 25163, Indonesia; 2 Department of Biology Education, STKIP YPM Bangko, Jambi 7314, Indonesia

**Keywords:** hydroxyapatite, alginate, composite, high thermal resistance

## Abstract

Hydroxyapatite (HAp) has been used for various applications such as orthopedics, drug delivery material, and bone tissue engineering. It is well known that HAp has a good biocompatibility and osteoconductivity, so HAp can be used in biomedical applications. Hydroxyapatite can be combined with other materials, in particular polymer, to expand its range of applications. In this study, the polymer that will be used as a support for the HAp composite is alginate (Alg). The HAp/Alg composite has been synthesized by the precipitation method. The XRD results show that the crystal system of HAp was hexagonal. The spheric-like shaped particles can be observed from SEM images, and particle size distribution spread from 400 to 1100 nm. The EDS spectrum exhibited the peak of Ca, C, P, and O elements, indicating that alginate had interacted with hydroxyapatite in the synthesized composite. The as-fabricated composite showed not only good crystallinity but also high thermal resistance. Thermogravimetric-differential thermal analysis (TGA-DTA) revealed that the HAp/Alg composites have a constant weight at 750 °C, so it might be applied in advanced applications such as bioimaging, drug carrier, and other cancer treatments.

## 1. Introduction

Hydroxyapatite (HAp), Ca_10_(PO_4_)_6_(OH)_2_ is a bioceramic-based calcium phosphate compound that is stable under body fluids [1]. The crystal structure of HAp is hexagonal, having a molar ratio of a Ca/P of 1.67 [2], which is currently widely applied in biomedical fields such as orthopedics and dentistry, bone tissue engineering, drug delivery materials, and cell imaging [3]. Various methods, such as the hydrothermal method [4], precipitation method [5], sol-gel method [6], electrodeposition techniques [7], double diffusion technique [8], biomimetics [9], emulsion [10] and template method [11] have reported for the fabrication of HAp. It can grow spheric-like, needle-like, fibrous, rod-shaped, irregularly spherical, and flake-shaped, depending on the different growth conditions of crystal faces of each method. 

However, to widen the application of HAp, it is mainly composited with other materials, one of them being a polymer. Polymers are large molecules formed by small and simple chemical units in the form of monomers. Therefore, polymers are often used, such as alginate [12], chitosan [13], cellulose [14], silk fibroin [1], PVA [15], and others. In this study, the polymer is alginate. Alginate is a polymer of great interest because it is an unbranched polysaccharide containing various compositions of beta-D-mannuronate (M) and alpha-L-guluronate (G) residues [12,13,14,15,16]. Hence the high cross-linking will reduce the hydrogel’s swelling and create a trapped drug. In addition, alginate (Alg) is not only cheap but also has good biocompatibility and biodegradability, so it supports drug delivery systems [2,17]. 

Recently, biopolymer matrix composites and inorganic reinforcement have been investigated for use in biomedical applications, including drug delivery, tissue engineering, enzyme immobilization, bioadsorption, and dentistry. In addition, these new inorganic-reinforced composites have demonstrated improved mechanical properties. Various biopolymer composite materials have been prepared in many drug delivery applications to enhance drug encapsulation/loading. They provide sustained release capabilities due to the synergistic action of inorganic materials and biopolymers in the composite matrix. Therefore, the enhanced characteristics of these biopolymer matrix-based composites can be further modified by varying the reinforcement type and ratio. Recently, various inorganic materials, such as carbon nanotubes (CNT), montmorillonite (MMT), calcium silicate (CS), β-tricalcium phosphate (β-TCP), and hydroxyapatite (HAp), have been used as reinforcement to alginate-based composites for use in drug delivery as shown in Table 1 [18]. The table shows that alginate composites with CNT, MMT, CS, β-TCP, and HAp can be applied as materials drug carriers, such as metronidazole, theophylline, bovine serum albumin, ofloxacin, irinotecan, venlafaxine HCl, diclofenac sodium, and vancomycin.

Meanwhile, research on composites using alginate not as a matrix but as a reinforcement has also been carried out. Sekar et al. (2022) reported the preparation of HAp/SA by coprecipitation method with maximum fluoride adsorption capacity of HAp, HAp/SA1, and HAp/SA4 was 10 mg/g, 35 mg/g, and 50 mg/g, respectively [19]. Sukhodub et al. (2018) presented the synthesizing HAp/Alg nanocomposites with swelling(%) and porosity values in HAp/Alg composites calcined at 1100 °C higher than pure Hap [20]. So, adding alginate to HAp is expected to increase the adsorption ability of composite when it is applied for drug delivery in the following research.

**Table 1:** The previous study about the alginate-based composite in drug delivery application.

No	Komposit	Obat	Ref.
1	CS-ALG	Metronidazole	[21]
2	CNT-ALG	Theophylline	[22]
3	core-shell CS-ALG	Bovine serum albumin	[23]
4	HAp-ALG	Ofloxacin	[24]
5	MMT-ALG	Irinotecan	[25]
6	MMT-ALG	Venlafaxine HCl	[26]
7	ALG-PVP- HAp	Diclofenac sodium	[27]
8	β-TCP-ALG	Vancomycin	[28]

In this study, we aim to perform the following: synthesize hydroxyapatite-alginate (HAp/Alg) composites by precipitation method and study the alginate concentration role in the properties of HAp/Alg composite. The composites prepared were analyzed by a series of characterization techniques such as X-ray diffraction (XRD), scanning electron microscopy (SEM with EDAX), Fourier infra-red spectroscopy (FTIR), and thermogravimetric-differential thermal analysis (TG-DTA). 

## 2. Materials and Methods

### 2.1. Materials

HAp/Alg composites are fabricated using the reagent of calcium oxide extracted from a natural source (bamboo shell, *Sollen spp.*) and alginate impression material (Medical Instruments Co., Ltd, Shanghai, China). Ammonia Solution 25% (NH_4_OH), Di-ammonium hydrogen phosphate ((NH_4_)_2_HPO_4_), and Nitric acid (HNO_3_) were analytical grade and purchased from Merck (KGaA, 64271, Darmstadt, Germany).

### 2.2. Fabrication of HAp/Alg Composites

The method of synthesizing HAp/Alg composites is the precipitation method. The diammonium hydrogen phosphate and calcium oxide powder were weighted in a molar ratio of 1.67, and five samples of alginate powder of 9.1, 16.7, 23.1, 28.6, 33.3 *w*/*w*% were taken. The alginate sample was dispersed in diammonium hydrogen phosphate of 50 mL at 80 °C in a magnetic stirrer at 250 rpm for two hours. Subsequently, the calcium oxide solution in nitric acid was mixed into the solution containing diammonium hydrogen phosphate and alginate at 300 rpm for two hours. The pH of the mixture was adjusted by adding ammonia until the pH was 10. Next, the precipitate was rinsed a few times to obtain an ammonia-free solution. At last, the sample was dried at 110 °C for about five hours to obtain HAp/Alg composites.

### 2.3. Characterizations

Calcium in bamboo shell powder was analyzed using X-ray fluorescence (PAN analytical). Then, the structural phase of the prepared composite was evaluated by X-ray diffraction (XRD) (PAN analytical, Malvern Panalytical, Malvern, UK) at 10–60° using radiation of Cu-Ka (k = 1.54 Å.) with a step size is 0.02°. The absorbance bands were revealed by Fourier Transform of Infra-Red spectroscopy (PerkinElmer Spectrum IR Version 10.6.1, Waltham, MA, USA) in the range of 4000–400 cm^−1^ using the ATR technique. The morphology and composition characterizations of the product were observed by SEM scanning electron microscope (Thermo Scientific Quatro S, Thermo Fisher Scientific, Waltham, MA, USA). The sample is sprinkled on carbon tape and then coated with gold. The coating method is ion sputerring. Then, the size particle distribution was measured using ImageJ software. The product’s thermal characteristic (10 mg) was evaluated by TG-DTA (Perkin Elmer) from 0 °C to 800 °C at a rate of 10 °C min^–1^.

## 3. Results and Discussion

### 3.1. The XRF Analysis

**Figure 1:**
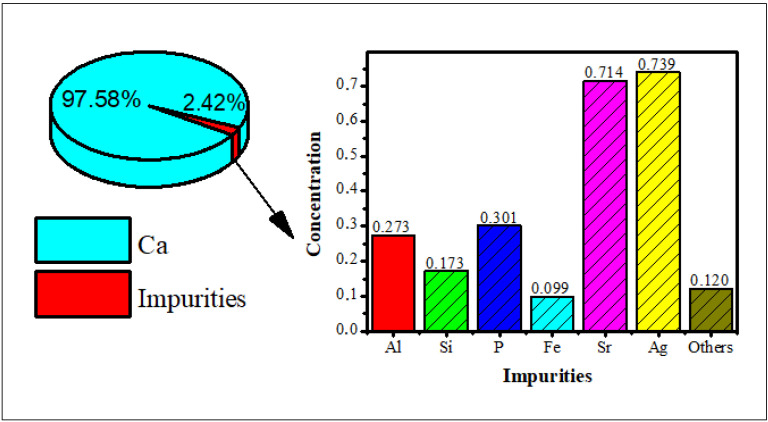
The X-ray fluorescence of bamboo shell *Sollen spp*.

Hydroxyapatite was synthesized using bamboo shell *Sollen spp.* as a biosource of calcium ions. The XRF analysis showed that the highest content of the shell is calcium (Figure 1), so it can be used as a precursor of calcium for the synthesis of the hydroxyapatite.

### 3.2. The Structural Analysis

**Figure 2:**
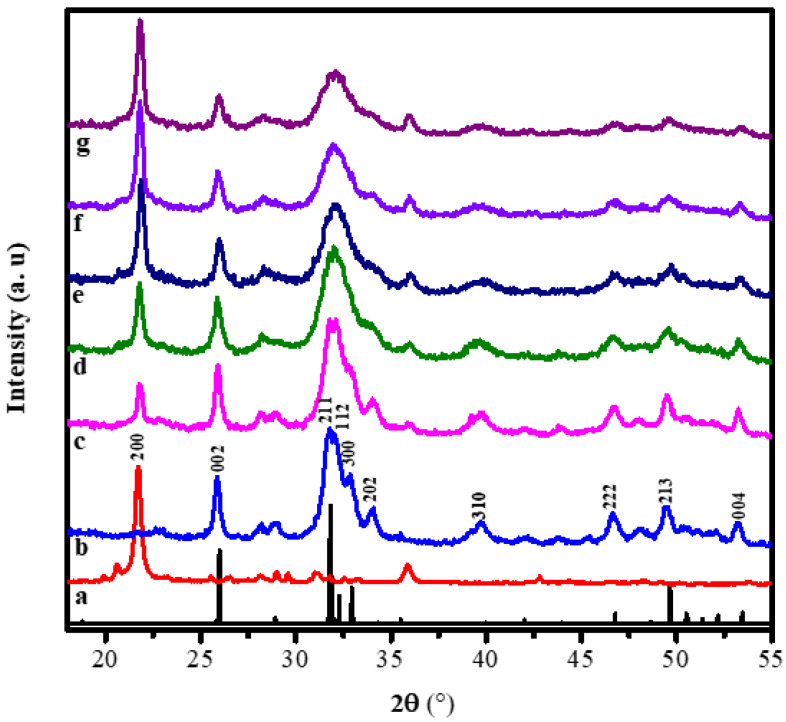
The XRD patterns of (**a**) the pure alginate, (**b**) the pure HAp, (**c**) HAp/Alg 9.1%, (**d**) HAp/Alg 16.7%, (**e**) HAp/Alg 23.1%, (**f**) HAp/Alg 28.6%, and (**g**) HAp/Alg 33.3%.

The diffraction patterns of the alginate, hydroxyapatite, and hydroxyapatite-alginate composites in some variations are displayed in Figure 2. Figure 2a,b exhibited pure alginate and hydroxyapatite diffraction patterns. The characteristic peak of alginate appeared at 21.71° belonged to the (200) crystal plane [14]. The characteristic peaks of HAp appeared at 25.96°, 31.86°, 32.16°, 32.46°, 34.08°, 39.86°, 46.78°, 49.48°, 53.28°, which belonged to (002), (211), (112), (300), (202), (310), (222), (213), and (004) crystal planes, respectively, according to ICSD number 157481 [29,30,31]. Most of the characteristic peaks of HAp and alginate appear in the diffraction pattern of composites, indicating the interaction between HAp and alginate in the composite. 

The Scherrer equation (Equation (1)) was used to determine the particles crystallite size.

D_hkl_ = 0.9·λ / β·cos θ (1)

where λ is the wavelength of X-rays (1.5406 Å), β is full width at half maximum (FWHM) value, and θ is the diffraction angle [32]. The crystallite sizes of HAp and all of the composite are displayed in Table 2. It can be observed that the crystallite size tends to increase as the concentration of alginate increases. The crystal size of HAp, HAp/Alg 9.1%, HAp/Alg 16.7%, HAp/Alg 23.1%, HAp/Alg 28.6%, and HAp/Alg 33.3% are 4.80 nm, 5.90 nm, 5.99 nm, 4.28 nm, 5.23 nm, and 5.60 nm, respectively.

### 3.3. The FTIR Analysis

**Figure 3:**
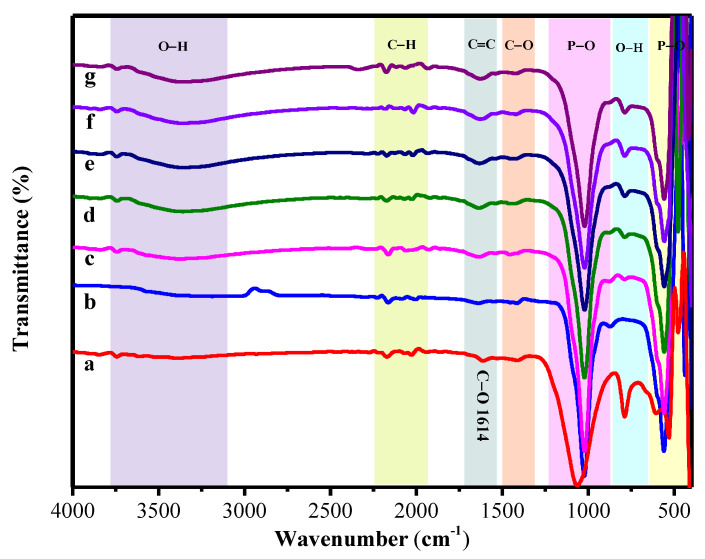
The FT-IR spectrum of (**a**) the pure alginate, (**b**) the pure HAp, (**c**) HAp/Alg 9.1%, (**d**) HAp/Alg 16.7%, (e) HAp/Alg 23.1%, (**f**) HAp/Alg 28.6%, and (**g**) HAp/Alg 33.3%.

The spectra of FTIR of the pure alginate, the hydroxyapatite, and the hydroxyapatite-alginate 9.1%, 16.7%, 23.1%, 28.6%, and 33.3% composite are displayed in Figure 3. The absorption bands are observed at 963; 438.56; 1023 and 560.64 cm^−1^ for symmetric stretching v_1_, bending v_2_, asymmetric stretching v_3_, and bending v_4_ of ${\mathrm{PO}}_{4}^{3-}$ group, respectively [13,33,34]. A broad band at 3359 cm^−1^ was marked to the O–H stretching vibration of hydroxyapatite and alginate in composite [35]. Meanwhile, the main characteristic bands of alginate involve two carboxylic group bands at 1613 cm^−1^ and 1421 cm^−1^ as (–COO– asymmetric) and (–COO– symmetric) bands [2,33]. Moreover, the vibration mode of C–O stretching appeared at 1421 cm^−1^ [36]. Mostly the absorption band is shifted toward the lower and higher region due to some interaction between hydroxyapatite and alginate in composite fabrication, as displayed in Table 3. However, these results correspond to the XRD result, as shown in Figure 2. 

### 3.4. Surface Morphologies and Chemical Compositions

**Figure 4:**
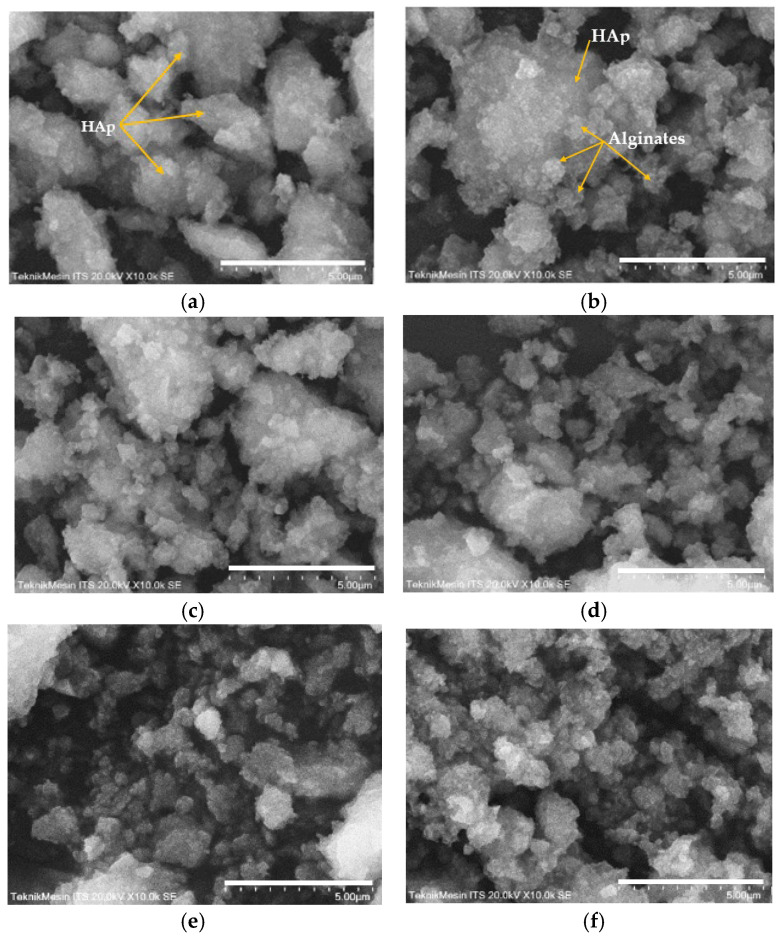
SEM images of (**a**) the pure HAp, (**b**) HAp/Alg 9.1%, (**c**) HAp/Alg 16.7%, (**d**) HAp/Alg 23.1%, (**e**) HAp/Alg 28.6%, and (**f**) HAp/Alg 33.3%.

**Figure 5:**
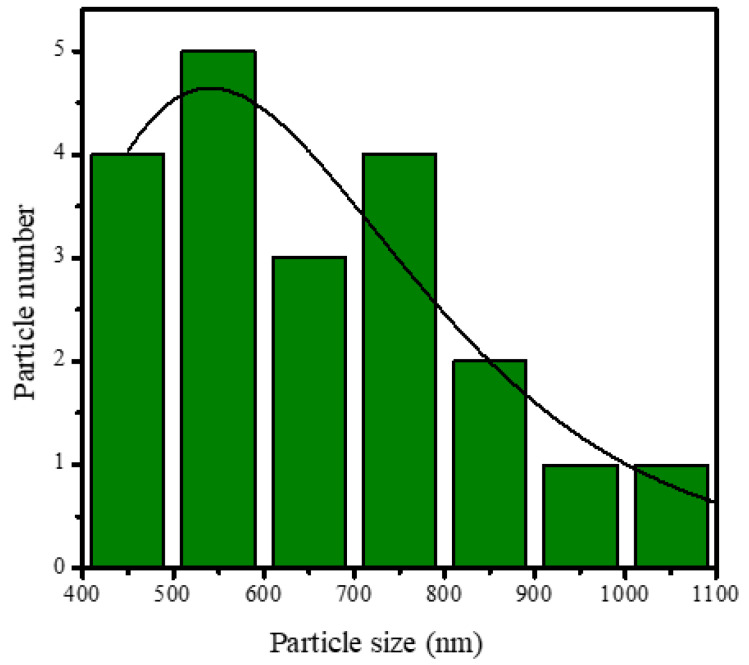
Particle size distribution of HAp/Alg 33.3% composite.

**Figure 6:**
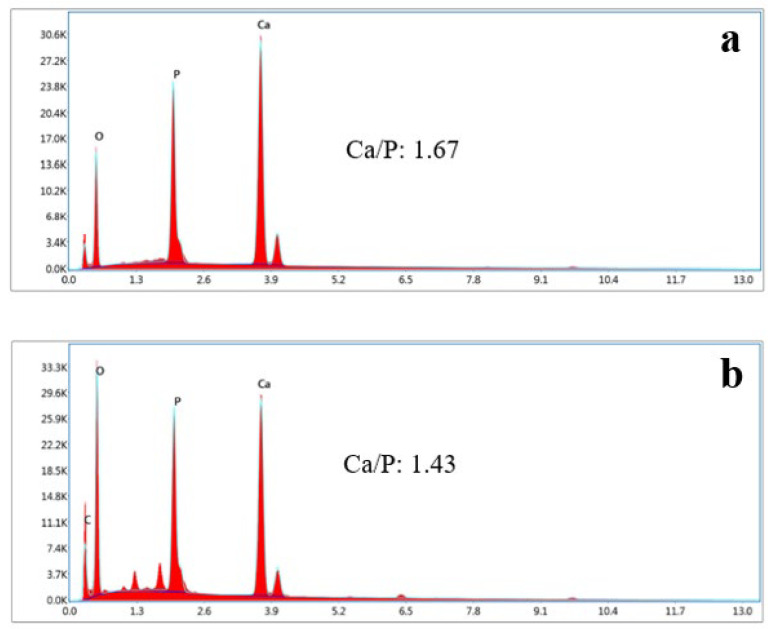
EDX spectrum of (**a**) the pure HAp, (**b**) HAp/Alg 33.3% composite.

SEM analysis of the HAp and HAp/Alg composites is demonstrated in Figure 4. Figure 4a is the SEM image of HAp, which shows that HAp has irregular flake-like shapes [37]; meanwhile, in Figure 4b, the alginate particles are well dispersed and distributed over the HAp particles [20]. However, due to the concentration of alginate increases the SEM images of HAp/Alg 28.6% and HAp/Alg 33.3% show spheric-like particles only with no flake-like particles. Among all variations in alginate concentration, the HAp/Alg 33.3% sample was more homogeneous in shape with a smaller particle size, which spread from 400 to 1100 nm, as shown in Figure 5. The EDS spectrum of HAp and HAp/Alg 33.3% are displayed in Figure 6, which exhibited the peak of Ca, P, and O elements for HAp and the peak of Ca, C, P, and O elements for HAp/Alg composite. The Ca/P molar ratio of HAp and HAp/Alg 33.3% composite are 1.67 and 1.43, respectively. The HAp/Alg 33.3% composite has a lower molar ratio because of the appearance of the C element from alginate. The attendance of the C element indicated that alginate had interacted with hydroxyapatite in the synthesized composite.

### 3.5. Thermal Stability

**Figure 7:**
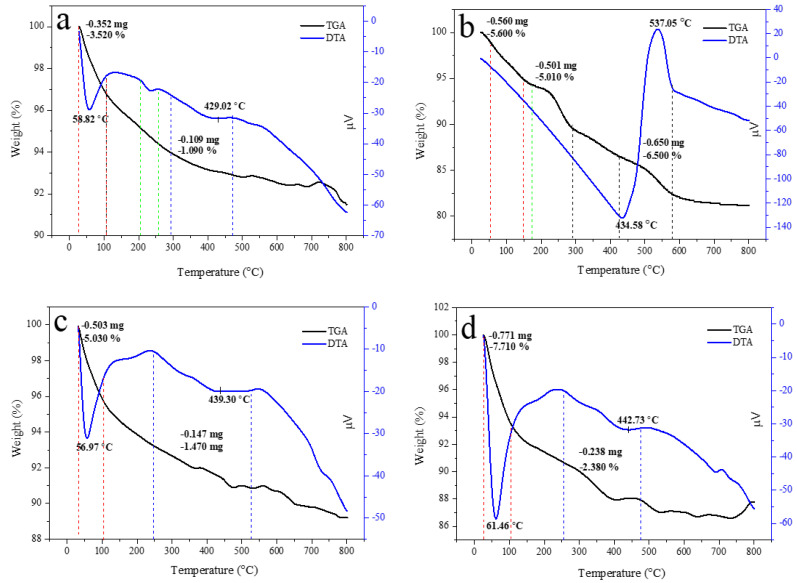

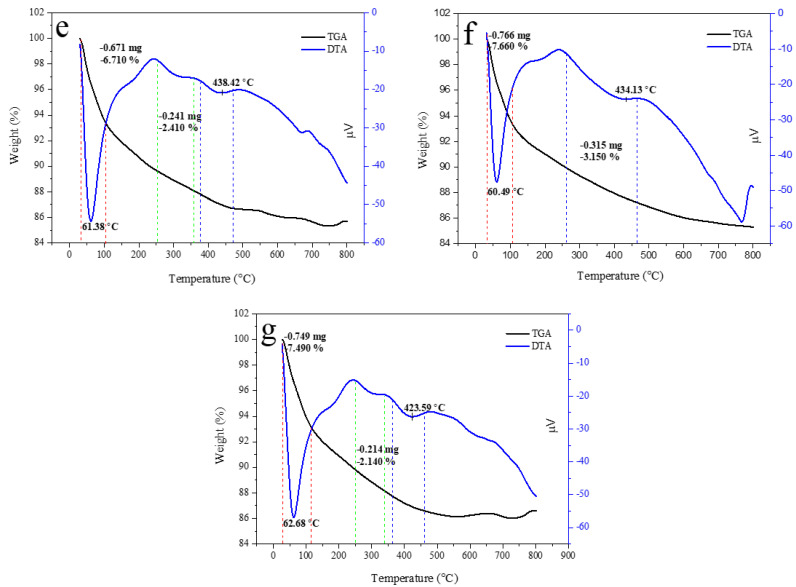
TGA/DTA curves of (**a**) the pure HAp, (**b**) the pure alginate, (**c**) HAp/Alg 9.1%, (**d**) HAp/Alg 16.7%, (**e**) HAp/Alg 23.1%, (**f**) HAp/Alg 28.6%, and (**g**) HAp/Alg 33.3%.

Thermal property is important for approximating the thermal performance and relative strength of the prepared sample. The TGA curve of HAp, alginate, and HAp/Alg composites was examined, as displayed in Figure 7. It shows a slow weight loss system between 30 and 750 °C. The TGA curve shows that the sample had a slight weight loss in the range of 30–150 °C could be due to water molecules vaporization in the prepared samples. The subsequent weight loss at the temperature between 200 and 350 °C is identified as the degradation of composite alginate reinforcement [33]. The last weight loss seems to be at 350 –500 °C, which might be attributed to the advanced degradation of the composite. Furthermore, at a temperature of about 600 °C, weight loss can be observed because of carbonation decomposition. As a result, there was no more weight loss up to 750 °C, which confirms the high thermal stability of the prepared composite [38]. 

Corresponding to the weight losses of the TGA analysis, the DTA curve presented sharp endothermic peaks at a minimum temperature of 58.82 °C for pure HAp, 56.97 °C for HAp/Alg 9.1%, 61.46 °C for HAp/Alg 16.7%, 61.38 °C for HAp/Alg 23.1%, 60.49 °C for HAp/Alg 28.6% and 62.68 °C for HAp/Alg 33.3%. On the contrary, the broad endothermic peaks were present at a minimum temperature of 434.58 °C for alginate. Moreover, the DTA curve displayed broad endothermic peaks at a minimum temperature of 429.02 °C for pure HAp, while 439.30 °C for HAp/Alg 9.1%, 442.73 °C for HAp/Alg 16.7%, 438.42 °C for HAp/Alg 23.1%, 434.13 °C for HAp/Alg 28.6% and 423.59 °C for HAp/Alg 33.3%. The maximum degradation rates of HAp/Alg composites were lower than those of pure HAp might be attributed to a strong interaction between alginate and HAp in the fabrication of the composite. 

The total percentage of weight losses during the whole process was about 4.61% for pure HAp, whereas 6.50% for the HAp/Alg 9.1%, 10.09% for the HAp/Alg 16.7%, 9.12% for the HAp/Alg 23.1%, 10.81% for the HAp/Alg 28.6% and 9.63% for the HAp/Alg 33.3%. These results imply that alginate plays a role in thermal stability improvement.

## 4. Conclusions

In this study, the HAp-Alg composites were successfully synthesized, and their structural phase, functional group, morphology, elemental composition, and thermal stability were explored. The XRD results show that the crystal system of HAp was hexagonal, and the crystallite size of HAp, HAp/Alg 33.3% was 4.80 nm; 5.60 nm, respectively. Moreover, the functional group, such as ${\mathrm{PO}}_{4}^{3-}$, OH^−^, COO, and C–H, were revealed by FTIR analysis. The porous spherical-shaped particles can be observed, and the distribution particle range of HAp/Alg 33.3% was spread from 400 to 1100 nm. Furthermore, the EDS spectrum of the synthesized composite is shown, which exhibited the peak of Ca, C, P, and O elements. The attendance of the C element indicated that alginate had interacted with hydroxyapatite in the synthesized composite. At last, the TGA-DTA thermogram shows no more weight loss up to 750 °C. It can be concluded that the prepared composite HAp/Alg has high thermal resistance, so it might be applied in many advanced applications such as bioimaging and drug carrying.

## Figures and Tables

**Table 2:** Comparison of crystallite sizes of various samples.

Sample	2θ (Degree) (211)	FWHM	Average Crystal Size (nm)
HAp	31.80	0.74009	4.80
HAp/Alg 9.10%	31.75	0.97988	5.90
HAp/Alg 16.7%	31.99	1.42138	5.99
HAp/Alg 23.1%	32.18	1.66924	4.28
HAp/Alg 28.6%	32.08	1.57521	5.23
HAp/Alg 33.3%	32.03	1.54234	5.60

**Table 3:** Functional group of the prepared sample.

Functional Group and Mode Vibration	Wavenumber (cm-1)						
	Alg	HAp	HAp/Alg 9.1%	HAp/Alg 16.7%	HAp/Alg 23.1%	HAp/Alg 28.6%	HAp/Alg 33.3%
Phosphate (PO_4_^3-^)							
*v_1_* symmetric stretching	-	963	963	963	963	963	963
*v_2_* bending	-	438.56	467.22	477.64	452.55	476.00	461.19
*v_3_* asymmetric stretching	-	1023.51	1022.49	1022.82	1022.18	1022.33	1022.34
*v_4_* bending	-	560.64 and 603	559.5 and 603	558.67 and 603	558.93 and 603	558.74 and 603	559.05 and 603
O–H stretching	-	3359	3364.37	3358.43	3357.47	3360.84	3348.93
C=C stretching		1640	1638.96	1637.35	1633.32	1629.33	1629.39
O–H of the carboxyl group	789	-	789.00	788.00	787.74	788.24	787.28
COO symmetric stretching	1421	-	1455	1446.5	1443	1424	1423
COO asymmetric stretching	1613	-	-	-	-	-	-
C–H stretching	2173	2164.50	2164.11	2175	2174	2186	2175.34

## Data Availability

Not applicable.

## References

[1] Mobika J., Rajkumar M., Nithya Priya V., Linto Sibi S.P. (2021). Effect of Chitosan Reinforcement on Properties of Hydroxyapatite/Silk Fibroin Composite for Biomedical Application. Phys. E Low-Dimens. Syst. Nanostructures.

[2] Adhikari J., Perwez M.S., Das A., Saha P. (2021). Development of Hydroxyapatite Reinforced Alginate–Chitosan Based Printable Biomaterial-Ink. Nano-Struct. Nano-Objects.

[3] Wulandari W., Wellia D.V., Jamarun N. (2021). The Effect of PH on the Synthesis and Characterization Hydroxyapatite from Bamboo Shell (*Sollen* Spp.) with Emulsion Method. J. Appl. Chem..

[4] Azis Y., Jamarun N., Arief S., Nur H. (2015). Facile Synthesis of Hydroxyapatite Particles from Cockle Shells (Anadaragranosa) by Hydrothermal Method. Orient. J. Chem..

[5] Jamarun N., Azharman Z., Arief S., Sari T.P., Asril A., Elfina S. (2015). Effect of Temperature on Synthesis of Hydroxyapatite from Limestone. Rasayan J. Chem..

[6] Layrolle P., Ito A., Tateishi T. (2005). Sol-Gel Synthesis of Amorphous Calcium Phosphate and Sintering into Microporous Hydroxyapatite Bioceramics. J. Am. Ceram. Soc..

[7] Shirkhanzadeh M. (1998). Direct Formation of Nanophase Hydroxyapatite on Cathodically Polarized Electrodes. J. Mater. Sci. Mater. Med..

[8] Kumar A.R., Kalainathan S. (2008). Growth and Characterization of Nano-Crystalline Hydroxyapatite at Physiological Conditions. Cryst. Res. Technol..

[9] Türk S., Altınsoy, ÇelebiEfe G., Ipek M., Özacar M., Bindal C. (2017). Microwave–Assisted Biomimetic Synthesis of Hydroxyapatite Using Different Sources of Calcium. Mater. Sci. Eng. C.

[10] Minaev N.V., Minaeva S.A., Sherstneva A.A., Chernenok T.V., Sedova Y.K., Minaeva E.D., Yusupov V.I., Akopova T.A., Timashev P.S., Demina T.S. (2022). Controlled Structure of Polyester / Hydroxyapatite Microparticles Fabricated via Pickering Emulsion Approach. Polymers.

[11] Bose S., Saha S.K. (2003). Synthesis and Characterization of Hydroxyapatite Nanopowders by Emulsion Technique. Chem. Mater..

[12] Ocando C., Dinescu S., Samoila I., Daniela Ghitulica C., Cucuruz A., Costache M., Averous L. (2021). Fabrication and Properties of Alginate-Hydroxyapatite Biocomposites as Efficient Biomaterials for Bone Regeneration. Eur. Polym. J..

[13] Jariya S.A.I., Padmanabhan V.P., Kulandaivelu R., Prakash N., Mohammad F., Al-Lohedan H.A., Paiman S., Schirhagl R., Hossain M.A.M., Sagadevan S. (2021). Drug Delivery and Antimicrobial Studies of Chitosan-Alginate Based Hydroxyapatite Bioscaffolds Formed by the Casein Micelle Assisted Synthesis. Mater. Chem. Phys..

[14] Zheng Y., Wang L., Bai X., Xiao Y., Che J. (2022). Bio-Inspired Composite by Hydroxyapatite Mineralization on (Bis)Phosphonate-Modified Cellulose-Alginate Scaffold for Bone Tissue Engineering. Colloids Surf. A Physicochem. Eng. Asp..

[15] Salim S.A., Loutfy S.A., El-Fakharany E.M., Taha T.H., Hussien Y., Kamoun E.A. (2021). Influence of Chitosan and Hydroxyapatite Incorporation on Properties of Electrospun PVA/HA Nanofibrous Mats for Bone Tissue Regeneration: Nanofibers Optimization and in-Vitro Assessment. J. Drug Deliv. Sci. Technol..

[16] Sikkema R., Keohan B., Zhitomirsky I. (2021). Alginic Acid Polymer-Hydroxyapatite Composites for Bone Tissue Engineering. Polymers.

[17] Rahyussalim A.J., Aprilya D., Handidwiono R., Whulanza Y., Ramahdita G., Kurniawati T. (2022). The Use of 3D Polylactic Acid Scaffolds with Hydroxyapatite/Alginate Composite Injection and Mesenchymal Stem Cells as Laminoplasty Spacers in Rabbits. Polymers.

[18] Hasnain M.S., Ahmed S.A., Behera A., Alkahtani S., Nayak A.K. (2020). Inorganic Materials–Alginate Composites in Drug Delivery.

[19] Sekar S., Eswaran S., Kolanthai E., Rajaram V., Kalkura N. (2022). Materials Today: Proceedings Enhanced Stability of Hydroxyapatite / Sodium Alginate Nanocomposite for Effective Fluoride Adsorption. Mater. Today Proc..

[20] Sukhodub L.F., Sukhodub L.B., Litsis O., Prylutskyy Y. (2018). Synthesis and Characterization of Hydroxyapatite-Alginate Nanostructured Composites for the Controlled Drug Release. Mater. Chem. Phys..

[21] Javadzadeh Y., Hamedeyazdan S., Adibkia K., Kiafar F., Zarrintan M.H., Barzegar-Jalali M. (2009). Evaluation of Drug Release Kinetics and Physico-Chemical Characteristics of Metronidazole Floating Beads Based on Calcium Silicate and Gas-Forming Agents. Pharm. Dev. Technol..

[22] Zhang X., Hui Z., Wan D., Huang H., Huang J., Yuan H., Yu J. (2010). Alginate Microsphere Filled with Carbon Nanotube as Drug Carrier. Int. J. Biol. Macromol..

[23] Wu C., Fan W., Gelinsky M., Xiao Y., Chang J., Friis T., Cuniberti G. (2011). In Situ Preparation and Protein Delivery of Silicate-Alginate Composite Microspheres with Core-Shell Structure. J. R. Soc. Interface.

[24] Roul J., Mohapatra R., Kumar Sahoo S. (2013). QR Code for Mobile Users Preparation, Characterization and Drug Delivery Behavior of Novel Biopolymer/Hydroxyapatite Nanocomposite Beads. Asian J. Biomed. Pharm. Sci..

[25] Iliescu R.I., Andronescu E., Ghitulica C.D., Voicu G., Ficai A., Hoteteu M. (2014). Montmorillonite-Alginate Nanocomposite as a Drug Delivery System—Incorporation and in Vitro Release of Irinotecan. Int. J. Pharm..

[26] Jain S., Datta M. (2016). Montmorillonite-Alginate Microspheres as a Delivery Vehicle for Oral Extended Release of Venlafaxine Hydrochloride. J. Drug Deliv. Sci. Technol..

[27] Hasnain M.S., Nayak A.K., Singh M., Tabish M., Ansari M.T., Ara T.J. (2016). Alginate-Based Bipolymeric-Nanobioceramic Composite Matrices for Sustained Drug Release. Int. J. Biol. Macromol..

[28] Seidenstuecker M., Ruehe J., Suedkamp N.P., Serr A., Wittmer A., Bohner M., Bernstein A., Mayr H.O. (2017). Composite Material Consisting of Microporous β-TCP Ceramic and Alginate for Delayed Release of Antibiotics. Acta Biomater..

[29] Abdulkareem M.H., Abdalsalam A.H., Bohan A.J. (2019). Influence of Chitosan on the Antibacterial Activity of Composite Coating (PEEK /HAp) Fabricated by Electrophoretic Deposition. Prog. Org. Coat..

[30] Wan F., Ping H., Wang W., Zou Z., Xie H., Su B.L., Liu D., Fu Z. (2021). Hydroxyapatite-Reinforced Alginate Fibers with Bioinspired Dually Aligned Architectures. Carbohydr. Polym..

[31] Ali A., Hasan A., Negi Y.S. (2022). Effect of Carbon Based Fillers on Xylan/Chitosan/Nano-HAp Composite Matrix for Bone Tissue Engineering Application. Int. J. Biol. Macromol..

[32] Prekajski Đorđević M., Maletaškić J., Stanković N., Babić B., Yoshida K., Yano T., Matović B. (2018). In-Situ Immobilization of Sr Radioactive Isotope Using Nanocrystalline Hydroxyapatite. Ceram. Int..

[33] Sirajudheen P., Karthikeyan P., Vigneshwaran S., Basheer M.C., Meenakshi S. (2021). Complex Interior and Surface Modified Alginate Reinforced Reduced Graphene Oxide-Hydroxyapatite Hybrids: Removal of Toxic Azo Dyes from the Aqueous Solution. Int. J. Biol. Macromol..

[34] Charlena, Bikharudin A., Wahyudi S.T. (2017). Erizal Synthesis and Characterization of Hydroxyapatite-Collagen-Chitosan (Ha/Col/Chi) Composite by Using Ex-Situ Wet Precipitation Method. Rasayan J. Chem..

[35] Bera M., Gupta P., Maji P.K. (2018). Facile One-Pot Synthesis of Graphene Oxide by Sonication Assisted Mechanochemical Approach and Its Surface Chemistry. J. Nanosci. Nanotechnol..

[36] Hoang V., Doan M., Mondal S., Mai T., Vo T., Duong C., Dat D., Nguyen V.T., Park S., Choi J. (2022). Colloids and Surfaces B: Biointerfaces Fluorescence Conjugated Nanostructured Cobalt-Doped Hydroxyapatite Platform for Imaging-Guided Drug Delivery Application. Colloids Surf. B Biointerfaces.

[37] Vázquez M.S., Estevez O., Ascencio-Aguirre F., Mendoza-Cruz R., Bazán-Díaz L., Zorrila C., Herrera-Becerra R. (2016). Tannic Acid Assisted Synthesis of Flake-like Hydroxyapatite Nanostructures at Room Temperature. Appl. Phys. A Mater. Sci. Process..

[38] Akter Jahan S., Mollah M.Y.A., Ahmed S., Abu Bin Hasan Susan M. (2017). Nano-Hydroxyapatite Prepared from Eggshell-Derived Calcium-Precursor Using Reverse Microemulsions as Nanoreactor. Mater. Today Proc..

